# Associations between treatment goals, patient characteristics, and outcome measures for patients with musculoskeletal disorders in physiotherapy practice

**DOI:** 10.1186/s12891-021-04048-4

**Published:** 2021-02-13

**Authors:** Olav Amundsen, Nina Køpke Vøllestad, Ingebrigt Meisingset, Hilde Stendal Robinson

**Affiliations:** 1 Institute of Health and Society, Faculty of Medicine, University of Oslo, P. O box 1130 Blindern, 0318 Oslo, Norway; 2 Department of Public Health and Nursing, Norwegian University of Science and Technology (NTNU), Trondheim, Norway

## Abstract

**Background:**

Goal setting is linked to person-centred care and is a core component in physiotherapy, but the associations between goal classes, patient characteristics and outcome measures for musculoskeletal disorders has not been investigated. The study’s purpose was to examine 1) how goals used in clinical practice for patients with musculoskeletal disorders (MSD) are distributed in classes based on ICF, 2) if goal classes were associated with patient characteristics and 3) whether goal classes were associated with treatment outcome.

**Methods:**

Data analysis from a longitudinal observational study (*N* = 2591). Goals were classified in symptom, function/structure, activity/participation and non-classifiable. Associations between patient characteristics and goal classes were examined using x^2^ and one-way ANOVA. Association between goal classes and outcomes were examined using multiple logistic and linear regression models. Outcomes are reported at 3 months or end of treatment if prior to 3 months.

**Results:**

There was a high variability in goals used for patients with MSD. 17% had symptom goals, 32.3% function/structure, 43.4% activity/participation and 7.4% non-classifiable goals. We found significant associations between goal classes and age, gender, severity, region of pain/diagnosis and emotional distress (all *p* < .001). Activity/participation goals were associated with better outcomes on GPE (OR 1.80, 95% CI 1.23–2.66). Non-classifiable goal was associated with poorer outcomes on pain intensity (B .87, 95% CI .32–1.43).

**Conclusion:**

There is an association between goal classes and patient characteristics. Including activity/participation in the main goal was associated with better outcomes for GPE and having a non-classifiable goal was associated with poorer outcomes for pain intensity.

**Trial registration:**

The project is approved by the Regional committee for Medical and Health Research Ethics in Norway (REC no. 2013/2030). https://clinicaltrials.gov/ct2/show/NCT03626389.

## Introduction

Musculoskeletal disorders (MSD) are a major social and economic burden and the leading cause of years lived with disability worldwide [1]. A systematic review of clinical guidelines for MSD endorse the use of person-centred care within a biopsychosocial model with the use of effective communication and shared decision-making, and recommend providing individualised care based on the persons context and preference [2]. The identification and setting of treatment goals with patients is clearly linked to person-centred care [3] and is regarded as a core component in the treatment process [4].

Previous research has shown that goals of physiotherapy interventions can be described using the The International Classification of Functioning, Disability and Health (ICF) [5]. One study in physiotherapy practice have indicated that person-centred meaningful treatment goals should correspond to the classification level of activity and participation in The International Classification of Functioning, Disability and Health (ICF) [3]. Furthermore, it is recommended to focus goal setting in clinical practice around behaviour related to activity and participation in ICF, rather than reporting of internal experiences, such as pain [6]. Studies of treatment goals used by patients with MSD show different results. Some studies have found that most patients define goals orientated towards activity, participation and coping [7] while others found higher use of symptom-focused goals [8–11]. The variation in goal classes might be explained by differences in patient characteristics and treatment settings, as there were important differences in age, employment, duration, diagnosis and recruitment settings between the studies.

Demographic, anthropometric, psychosocial and disease-specific factors are associated with outcomes for patients with MSD [12–19], and psychosocial factors have been shown to influence the goal setting process, engagement in goals, goal pursuit and goal conflicts [20–22]. Previous research has indicated that high symptom focus might prevent patients from reaching non-symptom related goals [9] and symptom distress is associated with less progression towards goals [23]. This suggests that a wide range of factors is important both for goal setting, goal pursuit and its relation to outcomes.

Previous studies have reported the association between goals, psychosocial factors [12–19] and their relation to outcomes [9, 23]. Despite this, we were not able to identify any studies that have compared the use of different goal classes and the associations between outcomes for patients with MSD. Furthermore, there is a lack of research on how patient characteristics are associated with treatment goals and how classifications of goals are associated with outcome measures.

We wanted to explore 1) how goals used in clinical practice for patients with MSD are distributed in classes based on domains of ICF, 2) if goal classes were associated with certain patient characteristics and 3) whether goal classes reflecting different domains of ICF were associated with treatment outcome.

## Methods

### Study design, setting and data collection

**Fig. 1:**
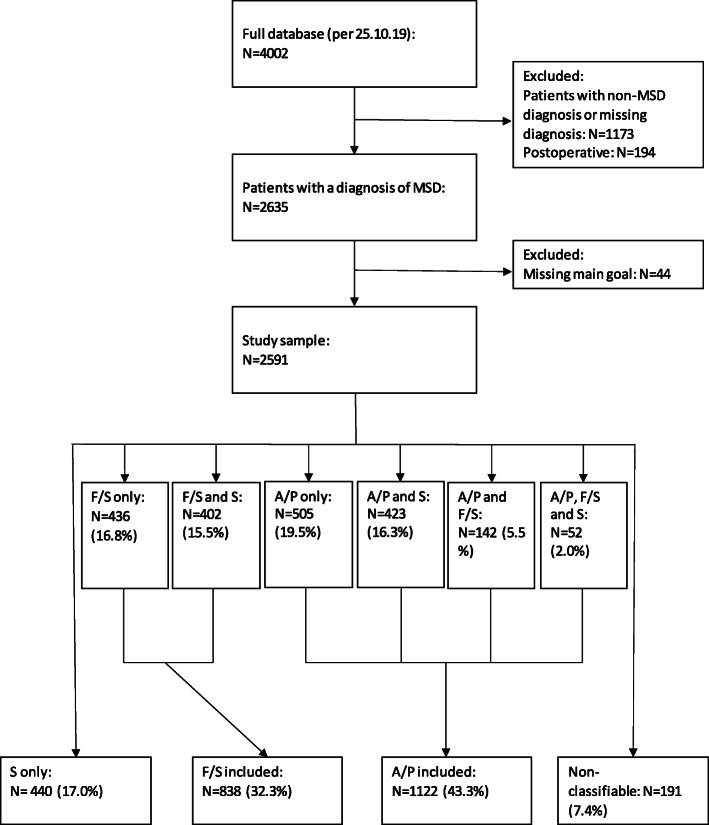
Flowchart showing inclusion of patients and categorizing of goal classes. MSD = Musculoskeletal disorders. S = Symptom. F/S = Function/structure. A/P = Activity/participation

This cohort study is part of the Norwegian longitudinal observational project Physiotherapy in Primary Health Care (FYSIOPRIM). The project includes data from adult patients seeking physiotherapy in nine municipalities Norway. All patients seeking physiotherapy in the 36 participating clinics in the primary health care system were eligible for inclusion. Patients were invited to participate on their first encounter with a physiotherapist in primary health care and were given project information and consent forms. The patients were treated by 109 different physiotherapists. 74 were general physiotherapists, 21 specialised as manual therapists and 14 specialised as psychomotor physiotherapists. Baseline data was collected in two steps. First, data were registered by the physiotherapist and the patient in collaboration. They jointly agreed, and defined, the main treatment goal and scored the most important functional problems using the Patient-Specific Functional Scale (PSFS) [24]. The physiotherapist registered diagnosis and determined whether the patient should fill in any disease- or region-specific questionnaires. Secondly, the patient completed questionnaires online. After 3 months or earlier if treatment was terminated before that, the physiotherapist and patient evaluated goal achievement and the patient completed the standardised outcome questionnaires. A more thorough description of the data collection for FYSIOPRIM and an overview of all recorded variables is presented in a previous article [25]. The full database included 4002 participants available for the present study, per 25.10.19. This study only included patients registered with a diagnosis related to the musculoskeletal system, excluding postoperative patients. A flowchart of the data collection process is presented in Fig. 1.

### Variables

For this study we included the following variables: treatment goal, body region/diagnosis, and demographic, anthropometric, psychosocial and disease specific variables previously shown to be associated to outcomes for MSD [12–19]. The demographic variables were age, gender and education (high school or less, up to four years of higher education or more than four years of higher education) and the anthropometric variable was BMI. Psychosocial variables were emotional distress, measured by Hopkins Symptom Checklist 10 (HSCL10), [26] and belief in effect of treatment measured by asking about agreement to the statement “I believe physiotherapy will improve my condition” (totally agree, agree, neither agree nor disagree, disagree or totally disagree). The disease-specific variables were duration of pain (first question on Short-Form Örebro; 0–1 weeks, 1–2 weeks, 3–4 weeks, 4–5 weeks, 6–8 weeks, 9–11 weeks, 3–6 months, 6–9 months, 9–12 months, over 1 year. Recoded to “less than 3 months”, “3–12 months” and “more than 12 months”) and severity (generated by average of pain last week (0–10, higher is more pain) and rating of the first activity on PSFS (0–10, higher is poorer self-rated ability on chosen activity).

The outcome measures were Global Perceived Effect 7-point scale (GPE) (Since the start of treatment, my current overall status is: 1 = very much better, 2 = much better, 3 = slightly better, 3 = neither better or worse, 5 = slightly worse, 6 = much worse, 7 = very much worse) [27], self-evaluated achievement of main goal (achieved, partly achieved or not achieved), change in PSFS and pain intensity after 3 months.

### Goal classes

Before statistical analysis could be conducted, the treatment goals were coded based on definitions of the goal being symptom related, function or structure related, activity or participation related, combinations of these or being non-classifiable. Definitions were based on the main categories from ICF [28], as follows: Function is defined as “physiological functions of body systems”. Structure is defined as “anatomical parts of the body such as organs, limbs and their components”. The definition of activity is “the execution of a task or action by an individual” and the definition for participation is “involvement in a life situation”. Symptoms such as pain are coded under body function in ICF. However, as previous research has suggested that symptom focused goals might affect outcome measures [9] and it has been recommended to not set goals related to internal experiences such as pain [6], we created a separate category directly related to symptoms. The goal classes were recoded, based on 1) goal including symptom, 2) goal including function or structure, 3) goal including activity or participation or 4) goal neither including activity, function nor symptom. This fourth class was termed “non-classifiable”. Patients without a defined treatment goal were excluded. The recoding of goals is illustrated in Fig. 1.

### Statistical method

Descriptive data are presented as frequencies and percentages or means and standard deviations as appropriate. Chi-square were used to examine univariable associations between categorical variables. One-way analysis of variance (ANOVA) with post hoc bonferroni test were used to examine associations between continuous variables.

To examine the associations between different goal classes and response on different treatment outcome measures, univariate logistic and linear regression analysis were used with 95% confidence intervals (CI). The assumptions of regression analysis were assessed. Multiple logistic and linear regression models were then used to control for demographic (age and gender), anthropometric (BMI), psychosocial (belief in effect of physiotherapy and emotional distress) and disease-specific variables (severity, pain duration and body region/diagnosis). All variables were examined in the univariate models. The variables that were statistically significant at the 20% level (*p* < 0.20) were included in the multiple regression models. A backward manual elimination method was used to remove those variables with the highest *p*-value. The elimination was repeated until only variables statistically significant at the 5% level (*p* < 0.05) remained. To prevent elimination of a variable at one step in the analysis process being crucial, the variables removed on backward elimination were all re-entered in the models one by one and remained in the models if they were statistically significant at the 5% level. Goal class, age and gender were kept in the multiple regression models regardless of significance.

To allow logistic regression, GPE and self-evaluated improvement on main goal were coded into a dichotomous scale. The GPE 7-point scale was divided into “improved” and “not improved”, where “improved” included the response of very much improved or much improved. “Not improved” included responses ranging from minimally improved to very much worse [27, 29]. For self-evaluated achievement of main goal, reports of main goal being achieved was categorized as “achieved”, and reports of main goal being partly achieved or not achieved was categorized as “not achieved”. Linear regression was used for PSFS and pain intensity. As we had a large database and the data material was already collected before this study started, a sample size analysis was not conducted. A common model to calculate sample size would be the formula *N* > 50 + 8 m (m = number of independent variables) for sample size calculations for multiple regressions. The lowest number of observations for one multiple regression analysis in our study was 975. This shows that the sample size was sufficient for the multiple regression analyses.

All statistical analysis was performed with IBM SPSS Statistics 25.

## Results

Figure 1 presents results from categorizing goals and the baseline characteristics of the total sample and the four goal classes are given in Table 1. Examples of goals from the dataset for activity/participation are “be able to walk and run as before injury”, “go hiking” and “get back to work” and examples of function/structure goals are “improved strength and range of motion” and “better knee function”. Symptom only goals examples are “pain reduction”, “pain relief” and “symptom absolution”. Examples of goals that could not be classified are “get well”, “get better” and “not getting worse”.

**Table 1:** Baseline patient characteristics and outcomes for the total study sample and for the four groups based on goal classes. Mean (SD) or N (%)

	Total ***N*** = 2591 (100%)	Symptom only ***N*** = 440 (17.0%)	Function/structure included ***N*** = 838 (32.3%)	Activity/participation included ***N*** = 1122 (43.3%)	Non-classifiable ***N*** = 191 (7.4%)	P
**Characteristics**						
Age years, mean (SD)	48.4 (18.1)	42.4 (16.9) ^bcd*^	52.0 (16.8) ^a^^c*^	47.5 (18.8) ^a*^	50.9 (17.9) ^a^^*^	**<.001**
Gender female, N (%)	1763 (69.5)	330 (76.7)	563 (68.1)	723 (66.5)	147 (77.4)	**<.001**
BMI, mean (SD)	26.6 (10.4)	25.5 (4.9)	27.3 (13.1)	26.6 (10.2)	25.8 (4.5)	.092
Severity (0–10) N (%)	5.3 (1.7)	5.0 (1.7) ^c*^	5.3 (1.6)	5.5 (1.7) ^a^^*^	5.4 (1.6)	**<.001**
Body region/diagnosis N (%)						**<.001**
*Spinal*	750 (28.9)	211 (47.7)	220 (25.8)	259 (23.5)	60 (30.0)	
*Upper extremities*	504 (19.4)	68 (15.4)	228 (26.7)	187 (17.0)	21 (10.5)	
*Lower extremities*	1118 (43.0)	107 (24.2)	344 (40.3)	595 (53.2)	72 (36.0)	
*Unspecific/ multisite conditions*	225 (8.7)	56 (12.7)	61 (7.2)	61 (5.5)	47 (23.5)	
Emotional distress (HSCL10, score 1–4), mean (SD)	1.62 (.51)	1.66 (.51) ^d*^	1.60 (.49) ^d*^	1.60 (.50) ^d*^	1.86 (.61) ^abc^^*^	**.001**
Treatment beliefs, N (%)	1776					**.021**
*Totally agree*	994 (56.0)	128 (46.2)	332 (59.0)	466 (57.5)	68 (54.4)	
*Agree*	658 (37.0)	120 (43.3)	194 (34.5)	299 (36.9)	45 (36.0)	
*Neither agree nor disagree*	122 (6.9)	29 (10.5)	36 (6.4)	45 (5.5)	12 (9.6)	
*Disagree*	2 (0.1)	0 (0)	1 (0.2)	1 (0.1)	0 (0)	
Duration, N (%)	1817					.575
*< 3 m*	386 (21.2)	77 (24.1)	119 (19.6)	162 (21.8)	28 (19.2)	
*3-12 m*	518 (28.5)	85 (26.6)	170 (28.0)	222 (29.9)	41 (28.1)	
*> 12 m*	913 (50.2)	158 (49.4)	319 (52.5)	359 (48.3)	77 (52.7)	
Education, N (%)	2072					.350
*High school or less*	826 (39.9)	124 (36.5)	276 (40.5)	374 (41.5)	52 (34.9)	
*Up to 4y higher seducation*	663 (32.0)	118 (34.7)	211 (31.8)	276 (30.6)	58 (38.9)	
*4y higher education sor more*	583 (28.1)	98 (28.8)	195 (28.6)	251 (27.9)	39 (26.2)	
**Outcome measures**						
Global perceived effect – Improved	900 (65.0)	122 (55.5)	290 (64.2)	429 (71.1)	59 (54.1)	**<.001**
Main goal achieved	439 (42.6)	64 (42.7)	123 (37.4)	201 (45.8)	51 (50.0)	.081
PSFS change (− 10 to 10)	2.94 (2.98)	2.61 (2.96)	3.05 (2.94)	3.13 (3.04) ^d*^	2.23 (2.7) ^c*^	**.022**
Pain intensity change (−10 to 10)	−1.12 (2.40)	− 1.19 (2.36) ^d*^	−0.98 (2.35)	−1.34 (2.41) ^d*^	−0.31 (2.43) ^ac*^	**<.001**

*M* Mean, *SD* Standard deviation, *HSCL10* Hopkins Symptom Check List 10 item version, higher is worse. Severity = average of pain intensity last week (0–10) and inverted rating of PSFS (0–10), higher is worse. y = years, m = months. P = X^2^/ANOVA. ^a^: compared to symptom, ^b^: compared to function/structure, ^c^: compared to activity/participation, ^d^: compared to non-classifiable. *: post hoc bonferroni ≤.05

### Differences in patient characteristics between goal classes

There were statistically significant differences between goal classes for the following baseline characteristics: age, gender, severity, body regions and emotional distress (all *p* < .001) (Table 1).

The group with symptom only goals had patients with younger age (p < .001 compared to all other goal classes), more females (10.3% more than EC), more patients with spinal pain (65.4% higher than EC) and more unspecific/multisite conditions (46.2% higher than EC). Fewer patients in this group totally agreed in that they believed in effect of physiotherapy for their condition (17.4% less than EC) and more patients neither agreed nor disagreed (52.6% higher than EC). The group including function/structure related goals had more patients with upper extremity pain (37.8% higher than EC). The group with activity/participation goals had more males (10.3% higher than EC), more patients with lower extremity pain (25.4% higher than EC) and less patients scoring neither agree nor disagree to belief in effect of physiotherapy for their condition (19.2% less than EC). The group with non-classifiable goals had more females (10.4% higher than EC), more patients with unspecific/multisite conditions (171.7% higher than EC) and higher emotional distress (*p* < .001 compared to other goal classes). This group also had more people scoring neither agree nor disagree to belief in effect of physiotherapy for their condition (39.5% higher than EC).

### Associations between treatment outcomes and goal classes

Table 2, 3, 4 and 5 shows the crude and adjusted associations between the four different treatment outcomes and goal classes together with patient characteristics. After 3 months, 1384 patients had filled out GPE and 1031 patients reported on achievement of treatment goal after. For PSFS, 975 patients reported after 3 months, while 1273 patients reported on pain intensity after 3 months.

**Table 2:** Crude and adjusted odds ratios with 95% confidence intervals (CI) for global perceived effect (GPE), dichotomized in improved (1-2) and not improved (3–7) and goal classes and demographical, anthropometric, psychosocial and disease-specific variables. Odds ratio > 1 indicates higher odds for reporting improved. *N* = 1384

Variable	Crude estimates OR (95% CI)	p	Multiple regression model OR (95% CI)	p
Goal classes		**<.001**		**.008**
*Symptom only*	Reference		Reference	
*Function/structure included*	1.44 (1.04 to 2.00)	**.030**	1.22 (.81 to 1.84)	.337
*Activity/participation included*	1.98 (1.44 to 2.72)	**<.001**	1.80 (1.23 to 2.66)	**.004**
*Non-classifiable*	.95 (.60 to 1.50)	.768	1.15 (.66 to 2.00)	.791
Age (years)	.99 (.99 to 1.00)	**.037**	.99 (.98 to 1.00)	.054
Gender (male is reference)	1.01 (.79 to 1.29)	.914	.82 (.62 to 1.09)	.112
BMI	1.00 (.99 to 1.02)	.592		
Treatment belief ^a^		**<.001**		**<.001**
*Totally agree*	Reference		Reference	
*Agree*	.44 (.34 to.57)	**<.001**	.49 (.37 to .64)	**<.001**
*Neither agree nor disagree*	.29 (.18 to.47)	**<.001**	.35 (.21 to .58)	**<.001**
Emotional distress (HSCL10, score 1–4),	.44 (.35 to.56)	**<.001**	.50 (.38 to .66)	**<.001**
Severity (score 0–10)	.95 (.88 to 1.01)	.108		
Duration		**<.001**		
*< 3 months*	Reference			
*3–12 months*	1.0 (.68 to 1.46)	.986		
*> 12 months*	.62 (.44 to.86)	**.004**		
Body region/diagnosis		**<.001**		**.**056
*Spinal*	Reference		Reference	
*Upper extremities*	1.46 (1.05 to 2.03)	**.026**	1.17 (.79 to 1.73)	.435
*Lower extremities*	1.34 (1.02 to 1.76)	**.034**	1.03 (.74 to 1.43)	.856
*Unspecific/multisite conditions*	.54 (.36 to.80)	**.003**	.59 (.37 to .94)	**.027**

*OR* Odds radio, *CI* Confidence intervals, *HSCL10* Hopkins Symptom Check List 10 item version. Severity = average of pain intensity last week (0–10) and inverted rating of PSFS (0–10), higher is worse

^a^: One patient answered disagree and one patient answered totally disagree. Data not presented in table due to low number

**Table 3:** Crude and adjusted odds ratios with 95% confidence intervals (CI) for self-evaluated improvement on main goal, dichotomized in improved (achieved main goal) and not improved (partly achieved or not achieved) and goal classes and demographical, anthropometric, psychosocial and disease-specific variables. Odds ratio > 1 indicates higher odds for reporting improved. *N* = 1031

Variable	Crude estimates OR (95% CI)	p	Multiple regression model OR (95% CI)	p
Goal classes		.082		.066
*Symptom only*	Reference		Reference	
*Function/structure included*	.80 (.54 to 1.19)	.272	.59 (.34 to 1.00)	.051
*Activity/participation included*	1.09 (.75 to 1.58)	.669	.87 (.53 to 1.45)	.611
*Non-classifiable*	1.34 (.81 to 2.23)	.252	1.14 (.58 to 2.24)	.708
Age	.99 (.99 to 1.00)	.112	1.00 (.99 to 1.01)	.968
Gender	1.33 (1.01 to 1.77)	**.040**	1.16 (.78 to 1.72)	.455
BMI	.99 (.97 to 1.01)	.176		
Treatment belief^a^		.076		
*Totally agree*				
*Agree*	.70 (.51 to .96)	**.024**		
*Neither agree nor disagree*	.58 (.31 to 1.08)	.085		
Emotional distress (HSCL10, score 1–4),	.51 (.38 to .69)	**<.001**	.58 (.40 to .85)	**.005**
Severity (score 0–10)	.84 (.77 to .91)	**<.001**	.88 (.80 to .99)	**.025**
Duration		**<.001**		**.009**
*< 3 m*	Reference		Reference	
*3-12 m*	.48 (.32 to .73)	**.001**	.54 (.33 to .86)	**.009**
*> 12 m*	.51 (.35 to .73)	**<.001**	.54 (.35 to .82)	**.004**
Body region/diagnosis		**.004**		
*Spinal*	Reference			
*Upper extremities*	.84 (.57 to 1.23)	.371		
*Lower extremities*	.74 (.55 to 1.00)	.050		
*Unspecific/multisite conditions*	.40 (.24 to .66)	**<.001**		

*OR* Odds radio, *CI* Confidence intervals, HSCL10 = Hopkins Symptom Check List 10 item version. Severity = average of pain intensity last week (0–10) and inverted rating of PSFS (0–10), higher is worse

^a^: Two patients answered disagree. Data not presented in table due to low number

**Table 4:** Crude and adjusted B-value with 95% CI for change in Patient Specific Functional Scale (PSFS) (− 10 to 10, higher is better) from baseline to 3 months, goal classes, demographical, anthropometric, psychosocial and disease-specific variables. B > 0 indicates better outcome. *N* = 975

Variable	Univariate regression B (95% CI)	p	Multiple regression model B (95% CI)	p
Goal classes				
*Symptom only*	Reference		Reference	
*Function/structure included*	.44 (−.12 to .99)	.126	.35 (−.25 to .96)	.249
*Activity/participation included*	.52 (.02 to 1.05)	.057	.16 (−.42 to .74)	589
*Non-classifiable*	−.51 (−1.31 to .30)	.220	−.30 (− 1.17 to .56)	.492
Age	−.02 (−.03 to .01)	**<.001**	−.03 (−.04 to −.02)	**<.001**
Gender	.64 (.23 to 1.05)	**.002**	.42 (−.02 to .86)	.061
BMI	−.01 (−.03 to.02)	.662		
Treatment belief^a^				
*Totally agree*	Reference		Reference	
*Agree*	−.54 (−.99 to −.08)	**.021**	−.37 (−.80 to −.05)	.082
*Neither agree nor disagree*	−1.22 (−2.10 to −.34)	**.006**	− 1.10 (− 1.90 to −.30)	**.007**
Emotional distress (HSCL10, score 1–4),	−.87 (− 1.28 to −.47)	**<.001**	− 1.39 (− 1.81 to −.97)	**<.001**
Severity (score 0–10)	.52 (.41 to.64)	**<.001**	.66 (.53 to .78)	**<.001**
Duration				
*< 3 m*	Reference			
*3-12 m*	.19 (−.63 to 1.02)	.650		
*> 12 m*	.44 (−.30 to 1.17)	.246		
Body region/diagnosis				
*Spinal*	Reference			
*Upper extremities*	.82 (.28 to 1.37)	**.003**		
*Lower extremities*	.45 (.01 to .90)	**.048**		
*Unspecific/multisite conditions*	−.44 (− 1.16 to .29)	.236		

*CI* Confidence intervals, *HSCL10* Hopkins Symptom Check List 10 item version. Severity = average of pain intensity last week (0–10) and inverted rating of PSFS (0–10), higher is worse

^a^: Two patients answered disagree and one patient answered totally disagree. Data not presented in table due to low number

**Table 5:** Crude and adjusted B-value with 95% CI for change in pain intensity (− 10 to 10, higher is worse) from baseline to 3 months, goal classes, demographical, anthropometric, psychosocial and disease-specific variables. B > 0 indicates poorer outcome. *N* = 1273

Variable	Univariate regression B (95% CI)	p	Multiple regression model B (95% CI)	p
Goal classes				
*Symptom only*	Reference		Reference	
*Function/structure included*	.21 (−.19 to .60)	.304	.29 (−.09 to .68)	.137
*Activity/participation included*	−.17 (−.54 to .21)	.383	.08 (−.30 to .45)	.676
*Non-classifiable*	.89 (.32 to 1.46)	**.002**	.87 (.32 to 1.42)	**.002**
Age	.01 (.01 to .02)	**.002**	.01 (.01 to .02)	**.001**
Gender	−.22 (−.51 to .06)	.128	−.17 (−.44 to .11)	.239
BMI	.01 (−.01 to .02)	.676		
Treatment belief^a^				
*Totally agree*	Reference			
*Agree*	.19 (−.11 to .49)	.218		
*Neither agree nor disagree*	−.01 (−.58 to .57)	.989		
Emotional distress (HSCL10, score 1–4),	.24 (−.03 to .51)	.082		
Severity (0–10)	−.43 (−.51 to −.36)	**<.001**	−.45 (−.53 to −.38)	**<.001**
Duration				
*< 3 months*	Reference			
*3–12 months*	−.21 (−.79 to .37)	.514		
*> 12 months*	−.08 (−.60 to .43)	.752		
Body region/diagnosis				
*Spinal*	Reference		Reference	
*Upper extremities*	−.25 (−.64 to .13)	.205	−.23 (−.60 to .14)	.228
*Lower extremities*	−.01 (−.33 to .31)	.953	−.04 (−.35 to .27)	.796
*Unspecific/multisite conditions*	.81 (.31 to 1.33)	**.002**	.91 (.41 to 1.39)	**<.001**

*CI* Confidence intervals, *HSCL10* Hopkins Symptom Check List 10 item version. Severity = average of pain intensity last week (0–10) and inverted rating of PSFS (0–10), higher is worse

^a^: Two patients answered disagree and one patient answered totally disagree. Data not presented in table due to low number

Compared to the goal class symptom, the goal class *Activity/participation* was associated with improvement on the GPE (OR = 1.80; 95% CI = 1.23–2.66) in the multiple logistic regression analysis*.* In addition, lower age, more positive treatment beliefs and lower emotional distress was associated with improvement, while having unspecific/multisite pain were associated with less improvement.

There were no association between goal classes and self-evaluated achievement of treatment goal in the univariate or multiple logistic regression analysis (Table 3). Lower emotional distress, lower severity and duration less than 3 months were significantly associated with achieved treatment goal.

Similarly, there were no association between goal classes and PSFS used as outcome (Table 4). In the multiple linear regression analysis lower age, being female, answering totally agree in belief of effect of physiotherapy, lower emotional distress, higher severity rating and having upper extremity pain compared to spinal pain were significantly associated with better scores on the PSFS.

When pain intensity was used as outcome, *non-classifiable goal* was associated with less improvement with a B-value of .87 (Table 5) in the multiple linear regression analysis. In addition, higher age, lower severity, and unspecific/multisite pain were significantly associated with less improvement.

## Discussion

We found high variability in what treatment goals were used for patients with MSD seeking physiotherapy in primary health care in Norway. We found an association between goal classes and the patient characteristics; age, gender, severity, body region/diagnosis and emotional distress. The group with symptom only goals had significantly younger age, more females, more patients with spinal pain and unspecific/multisite pain and also fewer patients having a high belief in the effect of physiotherapy. The group with function/structure goals had more patients with upper extremity pain. In the group of activity/participation goals there were more males, more patients with pain in their lower extremities and less patients with a lower belief in the effect of physiotherapy. The group with non-classifiable goals had more females, more patients with unspecific/multisite pain and significantly higher emotional distress. The group also had a lower belief in effect of physiotherapy.

Our results show that including activity and/or participation in the main goal was associated with better outcomes for GPE, while having a non-classifiable goal was associated with poorer outcomes for pain intensity. However, the effects were small.

### Goal classes for MSD

We found large variability of goals for patients with MSD when classifying goals based on the main domains of ICF and a symptom class. About half the goals could be classified into a single domain, while 7.4% of the goals were non-classifiable in our classifying system. This was mainly because of the goals being too broad and general. Our study allowed the goal setting process to be controlled by the therapist and patient alone without giving information on how the goals would be classified or how to conduct the goal setting process. This allowed the goal setting to be influenced mainly by therapist and patient characteristics. The high variability in goals were therefore as we expected as previous research indicates that both different therapist and patient characteristics are likely to influence goal setting [7, 9, 30].

Our findings are comparable to previous studies. We found that close to 30% of patients with spinal pain reported a main goal orientated around symptoms alone. Similar to our finding, Van Dulmen et al. (2016) found that more than 70% of patients with spinal pain chose reduction of pain as one of their goals for physiotherapy treatment. In contrast to this finding, Gardner et al. (2015) showed that patients with chronic low back pain tend to set goals orientated around activity, participation and coping, and not symptoms alone. This contrast might be due to difference in recruitment setting as their study predominantly recruited non-care seeking participants from the community. It could also be due to the goal setting process, as their study included giving the patients preliminary information on the chronic pain model, self-management tips and goal setting before the patient filled in their goals.

We found interesting differences in use of goal classes between different body regions/diagnosis. Patients with spinal pain chose symptom related goals more than other patients, while patients with unspecific/multisite conditions had more non-classifiable goals. Patients with upper- or lower extremity pain used more goals related to function/structure or activity/participation. Henry et al. (2017) found that patients with chronic musculoskeletal pain who were prescribed long-term opioids, prioritized symptom relief as the most important goal. Most patients in their study had several pain sites, with lower back being reported by 75% and a substantial proportion had comorbid mental health diagnoses. This is somewhat comparable to our findings as most patients in their study would correspond to our categories of spinal pain or unspecific/multisite conditions, although we do not have information about the patients use of pain medication. This supports our finding that patients with spinal pain or unspecific/multisite conditions more often define treatment goals related to symptom relief. Our findings show that patients with MSD related to the extremities uses more goals related to function/structure and activity/participation, less symptom related goals and have less non-classifiable goals. This might indicate that it is easier for patients to set goals related to specific functions and activities when they have MSD related to their extremities compared to having spinal pain or an unspecific/multisite condition.

### Goal classes and patient characteristics

It has been indicated that goals related to the activity and participation domain in the ICF, are the most meaningful and person-centred goals [3]. Still, only about 43% of the goals reported in our dataset included the activity and participation domain. One interpretation of this finding could be that in a population with large differences in patient characteristics, it is expected that the goals will vary as well. Indeed, our findings show that goal classes are associated with different patient characteristics such as age, gender, severity, body region/diagnosis and emotional distress. There is also support for this view in the literature as studies using different patient populations has found different results in terms of what goals they choose [7–10]. Further, a qualitative study on the meaning of recovery after a musculoskeletal injury found that patients summarised recovery either as complete symptom cessation and pain-free function or as return to function despite residual pain. Both the meaning of recovery and the expectations of recovery were influenced by several factors such as diagnosis, radiographic imaging, prior experiences, general health perceptions and own sense of resilience [31].

### Goal classes and outcomes

We found an association between goal classes and GPE and pain intensity as outcome measures. Moreover, activity or participation related goals were associated with better outcomes on GPE and non-classifiable goals were associated with poorer outcomes for pain intensity. Previous studies on other patient groups have found comparable results, with improved outcomes for patients having goals comparable to activity or participation [32–34]. They studied patients with stroke [32], depression [33] and vulvodynia [34], and suggested that associations with motivation, behaviour change and self-efficacy could be possible explanations. One study has found strong links between goal preferences and activity patterns for patients with widespread pain, and concluded that reinforcing achievement goals was recommended to improve chronic pain adaptation for patients with fibromyalgia [35]. Together with our findings, this shows there are associations between goal setting and outcomes for different patient groups and that this might be related to factors such as motivation, behaviour change and self-efficacy. Furthermore, this might indicate that a better understanding of goal setting and how this potentially could improve outcomes for MSD is important, and future research within this field is warranted.

We found that non-classifiable goals were associated to characteristics previously linked to poorer outcomes for MSD, such as being female, having high psychosocial distress and multisite or unspecific pain diagnosis [12–18]. Furthermore, we also found that non-classifiable goals were associated with poorer outcomes on pain intensity. This could be seen as an example of the multidimensional complexity of MSD and that a multitude of factors seem to be interlinked and might become barriers to improvement. Goal setting is considered important to plan interventions, direct attention and for motivational purposes [4, 36], and our findings show that the type of goal can reflect certain baseline characteristics. Emerging evidence supports that MSD needs to be evaluated in a broad perspective [37] and shows there are certain phenotypes with increased risk for poorer outcomes [38]. We should be aware that patient goals might reflect some of the characteristics associated to poorer outcomes recommended to screen for in patients with MSD. This aspect should be considered especially in patients with non-classifiable goals, as these were associated with known prognostic factors for poorer outcomes, such as high emotional distress and multisite or unspecific pain diagnosis [12–18].

Interestingly, there were no association between goals and outcomes on PSFS or self-evaluated improvement on main goal. It could be expected that having a symptom related goal would be associated to less improvement on PSFS, as previous research has indicated that the pursuit of pain-related goals might negatively influence non-pain related goals [9]. However, we found no association between goal class and change on PSFS. Neither were there any association on self-evaluated achievement on main goal.

### Clinical implications

Our results provide a broader understanding and more knowledge about patients’ personalized goals for treatment. We found associations between goals, patient characteristics and outcomes as well as large variations in types of goals being important to patients. This knowledge can be useful for therapists in making more person-centred treatment plans.

### Strengths and limitations

One strength of this study is the use of a large database including more than 2500 patients with MSD seeking physiotherapy in primary health care. This indicates that our results reflect clinical practice in primary health care in Norway.

There are also some limitations that should be considered. Firstly, there was a high number of dropouts that did not fill out outcomes at 3 months. 1207 patients did not fill out GPE at 3 months, 1560 patients for self-evaluated improvement on main goal, 1616 for PSFS and 1360 for pain intensity. Secondly, as the only instruction to the therapist was to set a main goal in collaboration with the patient, it is unknown whether there was an equal contribution in the goal setting process. Previous research has shown that there is a large variation in contribution between therapist and patient when setting treatment goals. Therapists with a biomedical treatment focus was associated with less patient involvement in goal setting compared with therapists with a biopsychosocial focus [30]. We do not have information on the therapist’s treatment focus or the involvement of patients in goal setting and have consequently not been able to control for these factors.

## Conclusion

We found large variability in goals used for patients with MSD seeking physiotherapy in primary health care in Norway. The content of goals were classified to the whole range of the domains of ICF, with only about one third restricted to either function or structure improvement or limitations in activity or participation. Most goals included a mix of domains. Less than 10% of the goals were not classifiable according to ICF. We found an association between goal classes and the patient characteristics; age, gender, severity, body region/diagnosis and emotional distress. Additionally, we found a weak association between goal classes and outcomes, where including activity and/or participation in the main goal was associated with better outcomes for GPE and having a non-classifiable goal was associated with poorer outcomes for pain intensity.

## Data Availability

The data that support the findings of this study are available from Tjenester for Sensitive Data (TSD) but restrictions apply to the availability of these data, which were used under license for the current study, and so are not publicly available. Data are however available from the authors upon reasonable request and with permission of University of Oslo and (TSD).
